# Supplementary material for: Nurses Response to the Physical and Psycho-Social Care Needs of Patients with COVID-19: A Mixed-Methods Study
Source: Healthcare (Basel). 2024 Jan 3;12(1):114. doi: 10.3390/healthcare12010114 (PMC10778578; doi:10.3390/healthcare12010114)
Supplement: Supplementary file 1 [file healthcare-12-00114-s001.zip › healthcare-2741324-supplementary.pdf]

Supplementary Materials S1: nurses interview guide

1. You worked in the intensive and sub-intensive care units for COVID 19 patients.
    a. Which are your memories of that period?

2. In your opinion, which were the needs of the patients you cared for?
    a. Could you give me some examples?

3. In your opinion, which were the most important needs?
    a. could you give me some examples?

4. What do you think it was important to do, as regards nursing care, for those patients?
    a. Was there something that made it easier?
    b. Could you give me some examples?
    c. Did you encounter a difficulty when you tried to do it?
    d. Could you describe some situations?

5. You achieve some good results with your interventions?
    a. Could you give me some examples?

6. From your perspective, what the patients could have wanted to receive different or in addition from the nurses staff?

7. Which was your experience with patient's relatives?
    a. Do you remember something?

8. Based on your experience, what would you have done or what would you be doing differently?

9. Any other suggestions or comments?

Supplementary Materials S2: patients interview guide

1. You got COVID19 and had to be hospitalized in the intensive or sub-intensive care unit ? (please specify which of the two)

2. What were your most important needs?

    a. Could you give me some examples?
    b. For which of those needs you were helped by the nurses?

3. Were you satisfied with their actions?

4. For which needs were you not helped by the nurses?

5. In your opinion, what could have been the reasons for this lack of support?

6. What were the things that helped you to feel better?

7. What were the main difficulties you encountered during your staying in the intensive care unit?

8. What would you have liked them to do?

9. Do you have some suggestions for the nurses team?

Supplementary Material S3: Legitimation criteria	
**Legitimation type**	**Strategies used**
Commensurability approximation legitimation	The research was conducted by experienced researchers in qualitative, quantitative, and mixed methods. The qualitative approach was used to investigate the emic perceptive and the quantitative methodology focused on etic views. The expert in mixed methods assured a balanced account of both views.
Inside-outside legitimation	Quantitative data from nurses' and patients' survey, quotes from patients and nurses interviews and data from patients records were summarized in the Joint Display. To explore the emic perspective (patients' and nurses' views) we used descriptive thematic analysis. To ensure inter-rater reliability two researchers independently coded the texts of the interviews.
Integration legitimation	The authors tried to integrate the quantitative and the qualitative data. This is shown in the Joint Display.
Paradigmatic legitimation	The research questions guided the researchers in choosing the most suitable methodologies to decide the phases of the study, and the data collection methods and analysis. We created two questionnaires, based on Cantarelli's model of care and we tested them both with patients and nurses. We used a validated tool (The Newcastle Satisfaction with Nursing Scale) to assess the perception of quality of nursing care with patients.
Sample integration legitimation	With regard to the quantitative data, we used all the members of the eligible populations, both for patients and nurses, which might not be representative for the general worldwide situation during Covid-19 pandemic. Our intent was not to achieve generalizability but to evaluate these variables in our context. For the qualitative phase, the sample was convenient and purposive.
Sequential legitimation	The research team agreed to use a mixed-method with an explanatory sequential design because each phase was used to inform the data to be collected in the next phase. It ensures a better understanding of the complexity of the healthcare environment during the Covid-19 pandemic.
Socio-political legitimation	The results of our study highlighted how, in this difficult context, nursing care was complex, multifaceted, and with a constant and competent technical-relational presence at the patients' bedside. Nurses showed how they can be very creative and resilient, and able to find coping strategies to deal with this extraordinary situation, although in the long term, high levels of tiredness were experienced. This study provides useful information for future situations like the Covid-19 pandemic and can be useful for management and health policies. Nurses were the backbone of the health systems globally during the pandemic, and therefore should be recognized and valued for their strategic role.
Weakness minimization legitimation	For the quantitative data we used a validated tool (The Newcastle Satisfaction with Nursing Scale), for the questionnaire developed from the Cantarelli's model of care, we conducted a pilot test to assess its acceptability. For the qualitative data we used Guba and Lincoln's criteria, and for data integration we used the joint display. To improve the reliability of data extraction from patients records, two researchers extracted the data independently and then compared their results to check the consistency. Through these methods we tried to minimize the limits of each approach, trying to achieve the added value of gaining further insight into the problem, which would have been difficult to achieve if two separate approaches had been used.

(Legitimation criteria, adapted from: Younas A, Parveen Rasheed S, Zeb H. Using legitimation criteria to establish rigor in sequential mixed-methods research. Nurse Res. 2020 Sep;28(3):44–51). [40].

Supplementary Materials S4. Clinical patient record analysis

Needs according to Cantarelli's Model	Needs recorded / total in clinical records	Needs identified	Nursing objectives / total in clinical records	Objectives identified	Actions/ total in clinical records	Actions identified	Evaluations / total in clinical records	Evaluations identified	Other interventions / total in clinical records	Other interventions identified
**Need for breathing**	(N=15)	Respiratory assessment (N=8)<br>Cough (N=6)<br>Dyspnea (N=5)<br>Desaturation (N=14)<br>Hemoptysis (N=1)<br>Bronchial secretions (N=1)	Explicit (N=1)<br>Not stated (N=1)<br>Implicit (N=13)	Maintain saturation > 90-92% (N=3)<br>Promote expectoration (N=2)<br>Reduce oxygen therapy (N=1)<br>Weaning from the ventilator and modulation of nocturnal ventilator supports to promote daytime respiratory autonomy (N=1)	(N=15)	Mobilization (pronation) (N=6)<br>Clearing the airways from secretions (airway suction, encouraging expectoration) (N=9)<br>Oxygen management (administration, equipment management, surveillance) (N=15)<br>Monitoring/checking (vital signs, blood gas analysis, biological tests) (N=15)<br>Breathing exercises (N=6)<br>Educational activity (N=4)<br>Use of scales (mMRC, Borg) (N=2)	(N=15)	Periodic and final (N=12)<br>Periodic (N=3)	Yes (N=12)<br>No (N=3)	Prono-supination (N=3)<br>Recording RR (N=6)<br>EWS scale (N=1)<br>Recruiting other professionals: physiotherapy (N=7)<br>speech therapists (N=1)<br>palliative care (N=2)
**Need for nutrition and hydration**	(N=9)	Loss of appetite (N=2)<br>Risk of impaired swallowing/dysphagia (N=2)<br>Nausea and/or vomiting (N=5)<br>Blood sugar alteration (N=1)	Implicit (N=7)<br>Explicit (N=0)<br>Not stated (N=8)	Recover spontaneous eating (N=1)<br>Ensure adequate hydration (N=1)	(N=15)	Checking nutrition (quantity and quality) (N=15)<br>Diet reassessment/ modification/ personalization (N=4)<br>Weight and/or height control (N=13)<br>Various tests and controls (N=9)<br>Ensuring hydration and/or fluid balance (N=11)	(N=14)	Periodic and final (N=6)<br>Periodic (N=8)	Yes (N=12)<br>No (N=3)	Recruiting other professionals: physiotherapy (N=2);<br>speech therapists (N=4);

		Weight loss (N=1)<br>Abdominal assessment (N=1)				NGT management and associated controls (N=5)<br>Control and/or management of nausea and vomiting (N=5)<br>Helping with meals (position, feeding, etc.) (N=9)<br>Management of enteral/parenteral nutrition (N=4)<br>Reserve treatment (antiemetic) (N=6)<br>Implementing recovery of autonomy (N=2)<br>Administering sugar orally (hypoglycemia) (N=1)				dietitians (N=7);<br>use of Kondrupp scale (N=3).
**Need for elimination**	(N=15)	Intestinal transit alteration: (constipation) (N=8)<br>Diarrhea (N=5)<br>Altered urinary elimination (N=2)<br>Dysuria and stranguria (N=3)<br>Abdominal assessment (N=5)<br>Heartburn (N=1)	Implicit (N=12)<br>Explicit (N=1)<br>Not stated (N=2)	Prevent bladder globe (N=1)<br>Fecal elimination control (N=1)<br>Ensure regular blood flow (N=1)	(N=15)	Various checks (on feces and urine) (N=11)<br>Function surveillance (N=12)<br>VC management (controls) (N=5)<br>Help with elimination (N=8)<br>Enemas (N=3)<br>Proposed laxative treatment (N=2)<br>Reserve treatment (laxatives) (N=10)<br>Changing incontinence aids (N=3)<br>Abdomen check (N=4)<br>Stimulated hydration (N=1)<br>Hot compresses (N=1)	(N=15)	Periodic and final (N=10)<br>Periodic (N=5)	Yes (N=2)<br>No (N=13)	Globular abdomen control (N=1)<br><br>Stoma therapist enrolment (N=1)
**Need for hygiene**	(N=7)	Integumentary evaluation (N=2)<br>skin alteration: erythema 1/15<br>- ear pressure wounds 1/15<br>- mucosal alteration, oral cavity dryness 1/15<br>- tracheostomy wound 1/15<br>- nostril pain 1/15	Implicit 6/15 -<br>explicit 0/15<br>- no 8/15		15/15	Perform partial or complete hygiene in bed 8/15 - Oral hygiene 7/15 - help with shower or other (hair, back, ...) 11/15 - help with dressing 4/15 - practice washing or dressing 3/15 - skin check and incontinence aids 8/15 - applying creams or medications 3/15 - use of scales (Braden, Norton) 2/15	12/15	Periodic and final 5/15<br>- periodic 7/15	Yes 5/15	Use of scales (Norton - Braden) 4/15 - consultation with an occupational therapist 2/15

**Need for movement**	(N=7)	Movement alteration (generic) 2/15 - Mobility evaluation (MI) 3/15 - Risk of falls 2/15 - Lower limb cramps 1/15 - asthenia 1/15	Implicit 4/15 - Explicit 1/15 - no 10/15	Recover autonomy and strength for daily life activities 1/15	13/15	Gradual mobilization 2/15 - passive mobilization in an armchair or in bed 8/15 - stimulate mobilization 4/15 – Accompanying movements 6/15 - observation and control 2/15 - interventions to prevent falls 1/15	10/15	Periodic and final 5/15 - Periodic 5/15	Yes 8/15	Use of scales (Schmid) 2/15 – Enroll physiotherapist 7/15
**Need for sleep and rest**	(N=3)	Sleep-wake alteration (insomnia) 2/15 - night surveillance 1/15	Implicit 5/15 – explicit 0/15 - No 10/15		9/15	Sleep-wake rhythm surveillance 7/15 - sleep quality control 1/15 - Reserve therapy 5/15 - active listening 1/15 - reduce night surveillance 1/15	7/15	Periodic and final 2/15 - periodic 5/15	No 15/15	
**Cardio-circulatory**	(N=15)	Alteration of blood pressure (6/15), Hemodynamic assessment (MI) (8/15), Alteration of body temperature (12/15), Neurological evaluation (MI) (5/15), Alteration of cardiac function (2/15), Teaching (Clexane) (1/15)	Explicit (0/15), Not stated (3/15), Implicit (12/15)	None identified	15/15	Monitoring of vital signs and cardiac rhythm (continuously or intermittently, ECG) (15/15), Neurological Surveillance (pupil control, GCS, monitoring of the state of consciousness, SAS scale) (8/15). Autonomous therapy management (catecholamines, infusions, therapy in reserve) (9/15). Therapeutic education (2/15). Other nursing actions (stimulate patient to hydrate, apply cold compresses, anti-Trendelenburg positioning, wearing elastic stockings) (6/15). Management of central and peripheral venous access (4/15), Clinical reasoning (co-relationship of clinical status-drugs-diuresis-fluid balance, prevention actions) (5/15)	15/15	Periodic and final (13/15), Periodic (2/15)	No (15/15)	None
**Safe environment**	(N=7)	Risk of falling (2/15), Risk of delirium (3/15), Altered mood (3/15), Falls (1/15)	Explicit (0/15), Not stated (10/15), Implicit (5/15)	None identified	15/15	Accompanying movement and supporting mobilization (7/15), Ensuring a safe environment (13/15), Verifying patient's identity (12/15), Ensuring safe therapy intake (4/15), Fall risk prevention (use of stairs, brochures) (7/15), Cognitive/emotional state	(10/15)	Periodic and final (5/15). Periodic (4/15)	Facilitating contact with relatives (1/15)	None

						interventions (active listening, providing instructions/information, reassurance, reporting and conversations in times of crisis, performing cognitive capacity exercises) (3/15)				
**Communication**	(N=8)	Conversation about treatment (5/15), Ethical discussion (signed orders to not resuscitate) (3/15), Interview with family (1/15), Change in clinical condition (1/15), Mood alterations (1/15)	Explicit (1/15), Not stated (9/15), Implicit (5/15)	Healing and returning home (1/15)	13/15	Conversations (about state of crisis, with family, on the treatment process, with other professionals) (11/15), Encouraging communication/interaction between patient and family member (5/15), Encourage communication (stimulate the patient, speaking valve, use of mother tongue) (5/15), Active listening (welcoming the need for communication, involving the patient) (5/15)	(5/15)	Periodic and final (2/15). Periodic 3/15)	Yes (2/15)	Requesting psychological consultation - Exit (1/15), Spiritual consultation (1/15)
**Therapeutic procedures**	(N=11)	Tegmental evaluation MI (6/15), Skin alteration (3/15), Pain (3/15), Sensory alteration (1/15)	Explicit (1/15), Not stated (6/15), Implicit (8/15)	Patient would like to resolve problem relating to abdominal pain (1/15)	14/15	Prevention of pressure wounds (surveillance of skin integrity, skin hydration - creams, air mattress, postural changes, monitoring of complications, use of Braden scale) (7/15), Management of lesions according to protocol (6/15), Pain management (use of VAS scale, management of reserve therapy) (8/15), Surveillance of therapy complications (2/15).	(12/15)	Periodic and final (9/15), Periodic (3/15)	No (15/15)	None
**Diagnostic procedures**	(N=0)	None	Not stated (15/15)	None identified	13/15	Laboratory tests (blood gas analysis, blood cultures, urine cultures) (9/15), ECG recording and rhythm interpretation (10/15), Oxygen weaning management (4/15), MI physical examination (2/15)	(10/15)	Periodic and final (6/15), Periodic (4/15)	No (15/15)	None
